# (*E*)-2-[4-(Diethyl­amino)­styr­yl]-1-ethyl­pyridinium iodide monohydrate

**DOI:** 10.1107/S160053681300528X

**Published:** 2013-02-28

**Authors:** Suchada Chantrapromma, Nawong Boonnak, Narissara Kaewmanee, Ching Kheng Quah, Hoong-Kun Fun

**Affiliations:** a Department of Chemistry, Faculty of Science, Prince of Songkla University, Hat-Yai, Songkhla 90112, Thailand; b Faculty of Traditional Thai Medicine, Prince of Songkla University, Hat-Yai, Songkhla 90112, Thailand; c X-ray Crystallography Unit, School of Physics, Universiti Sains Malaysia, 11800 USM, Penang, Malaysia; d Department of Pharmaceutical Chemistry, College of Pharmacy, King Saud University, PO Box 2457, Riyadh 11451, Saudi Arabia

## Abstract

In the title hydrated salt, C_19_H_25_N_2_^+^·I^−^·H_2_O, the 4-(diethyl­amino)­phenyl unit of the cation is disordered over two positions in a 0.847 (3):0.153 (3) ratio. The cation is twisted, with dihedral angles between the pyridinium and benzene rings of 11.25 (13) and 10.7 (8)° for the major and minor components, respectively. In the crystal, the three components are linked into a centrosymmetric 2:2:2 unit by O—H⋯I and C—H⋯O hydrogen bonds. π–π inter­actions with centroid–centroid distances of 3.5065 (7)–3.790 (9) Å are also present.

## Related literature

For background to and applications of amino­styrylpyridinium compounds, see: Chanawanno *et al.* (2010 ▶); Larnbert *et al.* (1996 ▶). For related structures, see: Fun *et al.* (2011*a* ▶,*b* ▶); Kaewmanee *et al.* (2010 ▶). For bond-length data, see: Allen *et al.* (1987 ▶).
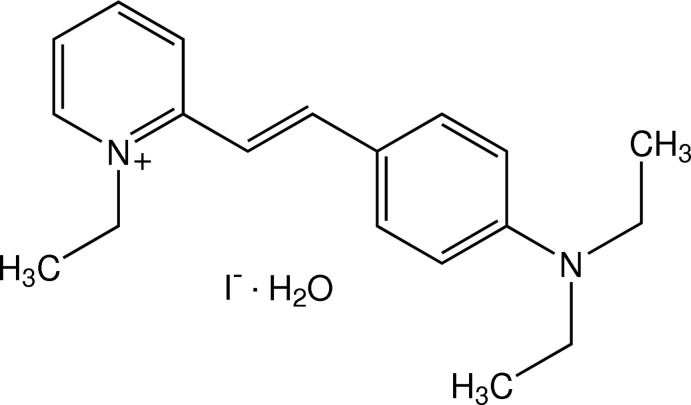


## Experimental

### 

#### Crystal data

C_19_H_25_N_2_^+^·I^−^·H_2_O
*M* *_r_* = 426.33
Triclinic, 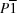

*a* = 7.9969 (1) Å
*b* = 9.1336 (1) Å
*c* = 14.7740 (2) Å
α = 96.220 (1)°
β = 105.430 (1)°
γ = 105.060 (1)°
*V* = 986.05 (2) Å^3^
*Z* = 2
Mo *K*α radiation
μ = 1.63 mm^−1^
*T* = 100 K
0.26 × 0.23 × 0.13 mm

#### Data collection

Bruker SMART APEXII CCD area-detector diffractometer
Absorption correction: multi-scan (*SADABS*; Bruker, 2009 ▶) *T*_min_ = 0.677, *T*_max_ = 0.816
32289 measured reflections
8664 independent reflections
7821 reflections with *I* > 2σ(*I*)
*R*_int_ = 0.025

#### Refinement

*R*[*F* ^2^ > 2σ(*F* ^2^)] = 0.025
*wR*(*F* ^2^) = 0.055
*S* = 1.08
8664 reflections
254 parameters
20 restraints
H atoms treated by a mixture of independent and constrained refinement
Δρ_max_ = 0.78 e Å^−3^
Δρ_min_ = −0.99 e Å^−3^

### 

Data collection: *APEX2* (Bruker, 2009 ▶); cell refinement: *SAINT* (Bruker, 2009 ▶); data reduction: *SAINT*; program(s) used to solve structure: *SHELXTL* (Sheldrick, 2008 ▶); program(s) used to refine structure: *SHELXTL*; molecular graphics: *SHELXTL*; software used to prepare material for publication: *SHELXTL* and *PLATON* (Spek, 2009 ▶).

## Supplementary Material

Crystal structure: contains datablock(s) global, I. DOI: 10.1107/S160053681300528X/is5238sup1.cif

Structure factors: contains datablock(s) I. DOI: 10.1107/S160053681300528X/is5238Isup2.hkl

Supplementary material file. DOI: 10.1107/S160053681300528X/is5238Isup3.cml

Additional supplementary materials:  crystallographic information; 3D view; checkCIF report

## Figures and Tables

**Table 1:** Hydrogen-bond geometry (Å, °)

*D*—H⋯*A*	*D*—H	H⋯*A*	*D*⋯*A*	*D*—H⋯*A*
O1*W*—H1*W*⋯I1^i^	0.85 (3)	2.71 (2)	3.5498 (12)	176 (2)
O1*W*—H2*W*⋯I1^ii^	0.86 (3)	2.75 (3)	3.6055 (13)	171 (2)
C3—H3*A*⋯O1*W* ^iii^	0.95	2.35	3.2072 (19)	150

Symmetry codes: (i) 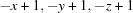; (ii) 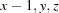; (iii) 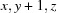.

**Fig. 1.**
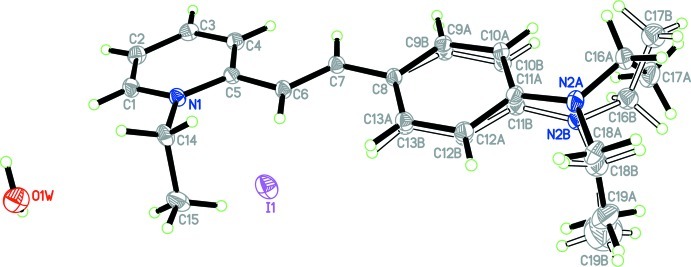
The asymmetric unit of the title compound, showing 50% probability displacement ellipsoids and the atom-numbering scheme. Open bonds show the minor component.

**Fig. 2.**
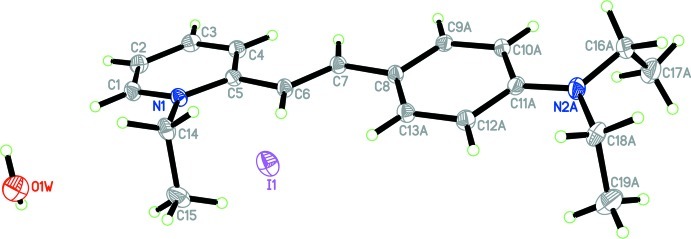
The molecular structure of the major component A, showing the orientation of the two ethyl groups of the diethylamino pointing towards the same direction.

**Fig. 3.**
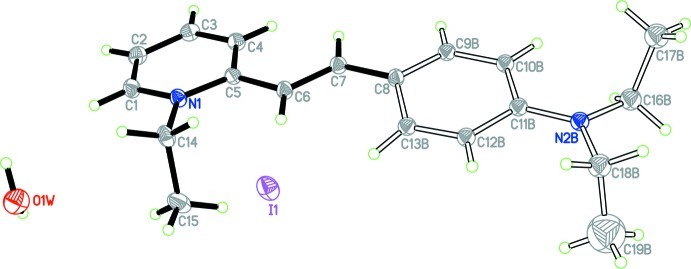
The molecular structure of the minor component B, showing the orientation of the two ethyl groups of the diethylamino pointing in opposite direction.

**Fig. 4.**
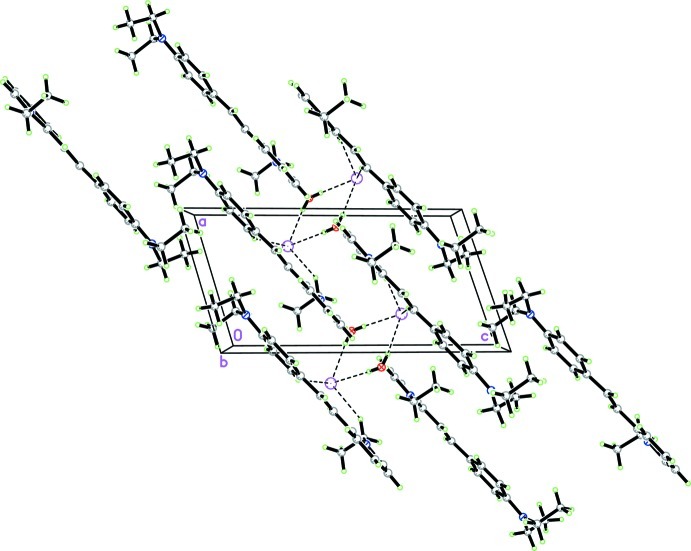
The crystal packing of the major component viewed along the b axis. Hydrogen bonds are drawn as dashed lines.

## supplementary crystallographic information

### Comment

Aminostyryl pyridinium (ASP) salts have been widely synthesized and used as fluorescence dyes for lymphocytes labeling and preliminary diagnostic imaging studies on dogs having a sodium-urate-induced inflammation in their stifle joints (Larnbert *et al.*, 1996). Moreover, ASP salts have been reported to possess antibacterial activity (Chanawanno *et al.*, 2010). During the course of our research on synthesis and antibacterial activity of quaternary ammonium compounds (Chanawanno *et al.*, 2010; Kaewmanee *et al.*, 2010), the title aminostyryl pyridinium derivative (I) was synthesized and tested for antibacterial activity. Our antibacterial assay showed that (I) exhibit moderate activity against *Pseudomonas aeruginosa*. Herein its crystal structure is reported.

The asymmetric unit of the title compound (I) (Fig. 1) consists of the C_19_H_25_N_2_^+^ cation, I^-^ anion and one H_2_O molecule. The 4-diethylaminophenyl unit of the cation is disordered over two positions; the major component *A* and the minor component *B* (Fig. 1), with the refined site-occupancy ratio of 0.847 (3)/0.153 (3). The cation exists in the *trans* configuration with respect to the C6═C7 double bond [1.3548 (16) Å] and the torsion angle C5—C6—C7—C8 = -176.54 (12)°. The cation is twisted as indicated by the dihedral angle between the C1–C5/N1 pyridinium and the C8–C13 benzene rings being 11.25 (13) and 10.7 (8)° for the major and minor components, respectively. It is interesting that the two ethyl groups of diethylamino moiety of both major and minor components deviated from the attached benzene ring but in different conformations in that the two ethyl units of the major component *A* point towards the same direction (Fig. 2), whereas they pointed opposite to each other for the minor component *B* (Fig. 3). These orientations of the diethylamino group can be indicated by the torsion angles C11A—N2A—C16A—C17A = -81.12 (19)° and C11A—N2A—C18A—C19A = 81.4 (2)° for the major component *A* and C11B—N2B—C16B—C17B = 87.0 (11)° and C11B—N2B—C18B—C19B = 93.0 (19)° for the minor component *B*. The other ethyl unit attached to atom N1 also deviated from its bound pyridinium ring with the torsion angle C5—N1—C14—C15 = -86.95 (14)° for the major component *A* and -81 (7)° for the minor component *B*. The bond lengths of cation are in normal ranges (Allen *et al.*, 1987) and comparable with related structures (Fun *et al.*, 2011*a*,*b*; Kaewmanee *et al.*, 2010).

In the crystal packing, the cations, anions and water molecules are linked into a centrosymmetric 2:2:2 unit of the three components by O—H···I hydrogen bonds and a weak C—H···O interaction (Fig. 4 and Table 1). π–π interactions with the centroid distances of *Cg*1···*Cg*1^v^ = 3.5065 (7) Å [symmetry code (v) = 1 - *x*, 2 - *y*, 1 - *z*], *Cg*1···*Cg*2^ii^ = 3.5796 (16) Å and *Cg*1···*Cg*3^ii^ = 3.790 (9) Å were observed; *Cg*1, *Cg*2 and *Cg*3 are the centroids of N1/C1–C5, C8/C9A–C13A and C8/C9B–C13B rings, respectively.

### Experimental

The title compound (I) was prepared by mixing 1:1:1 molar ratio solutions of 1-ethyl-2-methylpyridinium iodide (1 g, 4 mmol), 4-diethylaminobenzaldehyde (0.7 g, 4 mmol) and piperidine (0.4 ml, 4 mmol) in methanol (40 ml). The resulting solution was stirred for 4 h under a nitrogen atmosphere. The orange solid which formed was filtered and washed with diethylether. Orange block-shaped single crystals of (I) suitable for *x*-ray structure determination were recrystallized from methanol by slow evaporation at room temperature over a few weeks, M.p. 446–448 K.

### Refinement

Water H atoms were located in a difference Fourier map and the positions were refined freely, with *U*_iso_(H) = 1.5*U*_eq_(O). The remaining H atoms were fixed geometrically and all hydrogen atoms were allowed to ride on their parent atoms, with d(C—H) = 0.95 for aromatic and CH, 0.99 for CH_2_ and 0.98 Å for CH_3_ atoms. Their *U*_iso_ values were constrained to be 1.5*U*_eq_(C) for methyl H atoms and 1.2*U*_eq_(C) for the remaining H atoms. A rotating group model was used for the methyl groups. The 4-diethylaminophenyl unit is disordered over two sites with refined site occupancies of 0.847 (3) and 0.153 (3). The non-hydrogen atoms of the minor component were refined isotropically with the *U*_iso_ values of N2B, C9B, C10B, C11B, C12B and C13B being fixed at 0.01583 Å^2^.

### Crystal data

**Table:**

C_19_H_25_N_2_^+^·I^−^·H_2_O	*Z* = 2
*M**_r_* = 426.33	*F*(000) = 432
Triclinic, *P*1	*D*_x_ = 1.436 Mg m^−^^3^
Hall symbol: -P 1	Melting point = 466–468 K
*a* = 7.9969 (1) Å	Mo *K*α radiation, λ = 0.71073 Å
*b* = 9.1336 (1) Å	Cell parameters from 8664 reflections
*c* = 14.7740 (2) Å	θ = 1.5–35.2°
α = 96.220 (1)°	µ = 1.63 mm^−^^1^
β = 105.430 (1)°	*T* = 100 K
γ = 105.060 (1)°	Block, orange
*V* = 986.05 (2) Å^3^	0.26 × 0.23 × 0.13 mm

### Data collection

**Table:**

Bruker SMART APEXII CCD area-detector diffractometer	8664 independent reflections
Radiation source: fine-focus sealed tube	7821 reflections with *I* > 2σ(*I*)
Graphite monochromator	*R*_int_ = 0.025
Detector resolution: 8.33 pixels mm^-1^	θ_max_ = 35.2°, θ_min_ = 1.5°
ω scans	*h* = −12→12
Absorption correction: multi-scan (*SADABS*; Bruker, 2009)	*k* = −14→14
*T*_min_ = 0.677, *T*_max_ = 0.816	*l* = −23→23
32289 measured reflections	

### Refinement

**Table:**

Refinement on *F*^2^	Primary atom site location: structure-invariant direct methods
Least-squares matrix: full	Secondary atom site location: difference Fourier map
*R*[*F*^2^ > 2σ(*F*^2^)] = 0.025	Hydrogen site location: inferred from neighbouring sites
*wR*(*F*^2^) = 0.055	H atoms treated by a mixture of independent and constrained refinement
*S* = 1.08	*w* = 1/[σ^2^(*F*_o_^2^) + (0.0222*P*)^2^ + 0.355*P*] where *P* = (*F*_o_^2^ + 2*F*_c_^2^)/3
8664 reflections	(Δ/σ)_max_ = 0.002
254 parameters	Δρ_max_ = 0.78 e Å^−^^3^
20 restraints	Δρ_min_ = −0.99 e Å^−^^3^

### Special details

**Table:**

Experimental. The data was collected with the Oxford Cyrosystem Cobra low-temperature attachment.
Geometry. All e.s.d.'s (except the e.s.d. in the dihedral angle between two l.s. planes) are estimated using the full covariance matrix. The cell e.s.d.'s are taken into account individually in the estimation of e.s.d.'s in distances, angles and torsion angles; correlations between e.s.d.'s in cell parameters are only used when they are defined by crystal symmetry. An approximate (isotropic) treatment of cell e.s.d.'s is used for estimating e.s.d.'s involving l.s. planes.
Refinement. Refinement of *F*^2^ against ALL reflections. The weighted *R*-factor *wR* and goodness of fit *S* are based on *F*^2^, conventional *R*-factors *R* are based on *F*, with *F* set to zero for negative *F*^2^. The threshold expression of *F*^2^ > σ(*F*^2^) is used only for calculating *R*-factors(gt) *etc*. and is not relevant to the choice of reflections for refinement. *R*-factors based on *F*^2^ are statistically about twice as large as those based on *F*, and *R*- factors based on ALL data will be even larger.

### Fractional atomic coordinates and isotropic or equivalent isotropic displacement parameters (Å^2^)

**Table:**

	*x*	*y*	*z*	*U*_iso_*/*U*_eq_	Occ. (<1)
I1	0.748096 (12)	0.409068 (8)	0.331067 (6)	0.02160 (3)	
O1W	0.11907 (16)	0.28996 (13)	0.46705 (9)	0.0284 (2)	
H1W	0.155 (3)	0.359 (3)	0.5168 (17)	0.043*	
H2W	0.029 (3)	0.310 (3)	0.4293 (17)	0.043*	
N1	0.35581 (14)	0.74599 (11)	0.39233 (7)	0.01566 (17)	
C1	0.22228 (17)	0.76646 (14)	0.42876 (9)	0.0186 (2)	
H1A	0.1520	0.6823	0.4484	0.022*	
C2	0.18657 (17)	0.90514 (14)	0.43787 (9)	0.0197 (2)	
H2A	0.0926	0.9176	0.4632	0.024*	
C3	0.29171 (17)	1.02765 (14)	0.40898 (9)	0.0191 (2)	
H3A	0.2723	1.1260	0.4160	0.023*	
C4	0.42409 (17)	1.00497 (13)	0.37018 (9)	0.0175 (2)	
H4A	0.4942	1.0884	0.3498	0.021*	
C5	0.45769 (16)	0.86130 (12)	0.36008 (8)	0.01505 (19)	
C6	0.59165 (16)	0.83175 (13)	0.31664 (9)	0.0161 (2)	
H6A	0.5979	0.7292	0.3053	0.019*	
C7	0.70797 (16)	0.94503 (13)	0.29176 (8)	0.01576 (19)	
H7A	0.7022	1.0469	0.3076	0.019*	
C8	0.83991 (16)	0.92867 (12)	0.24387 (8)	0.01542 (19)	
C14	0.38311 (18)	0.59060 (13)	0.38875 (10)	0.0191 (2)	
H14A	0.3470	0.5438	0.4407	0.023*	
H14B	0.5137	0.6016	0.3994	0.023*	
C15	0.2723 (2)	0.48452 (15)	0.29321 (11)	0.0260 (3)	
H15A	0.2873	0.3816	0.2947	0.039*	
H15B	0.3145	0.5264	0.2421	0.039*	
H15C	0.1435	0.4771	0.2812	0.039*	
N2A	1.2553 (2)	0.91163 (15)	0.11947 (12)	0.0199 (3)	0.847 (3)
C9A	0.9548 (3)	1.0628 (4)	0.22893 (14)	0.0158 (4)	0.847 (3)
H9A	0.9404	1.1600	0.2487	0.019*	0.847 (3)
C10A	1.0880 (2)	1.0578 (2)	0.18638 (12)	0.0167 (3)	0.847 (3)
H10A	1.1620	1.1511	0.1770	0.020*	0.847 (3)
C11A	1.1162 (2)	0.9159 (2)	0.15659 (11)	0.0162 (3)	0.847 (3)
C12A	0.9984 (7)	0.7803 (5)	0.1698 (5)	0.0183 (9)	0.847 (3)
H12A	1.0092	0.6826	0.1477	0.022*	0.847 (3)
C13A	0.8679 (6)	0.7869 (6)	0.2142 (4)	0.0168 (7)	0.847 (3)
H13A	0.7954	0.6941	0.2249	0.020*	0.847 (3)
C16A	1.3859 (2)	1.05464 (18)	0.11576 (11)	0.0219 (3)	0.847 (3)
H16A	1.4992	1.0323	0.1127	0.026*	0.847 (3)
H16B	1.4162	1.1291	0.1759	0.026*	0.847 (3)
C17A	1.3210 (3)	1.1305 (2)	0.03204 (13)	0.0289 (4)	0.847 (3)
H17A	1.4122	1.2292	0.0378	0.043*	0.847 (3)
H17B	1.2056	1.1488	0.0324	0.043*	0.847 (3)
H17C	1.3031	1.0625	−0.0280	0.043*	0.847 (3)
C18A	1.2781 (3)	0.7663 (2)	0.08186 (18)	0.0221 (4)	0.847 (3)
H18A	1.2523	0.6923	0.1243	0.027*	0.847 (3)
H18B	1.4067	0.7846	0.0838	0.027*	0.847 (3)
C19A	1.1561 (5)	0.6927 (4)	−0.02058 (19)	0.0335 (5)	0.847 (3)
H19A	1.1788	0.5957	−0.0405	0.050*	0.847 (3)
H19B	1.1832	0.7635	−0.0637	0.050*	0.847 (3)
H19C	1.0282	0.6718	−0.0231	0.050*	0.847 (3)
N2B	1.1981 (11)	0.9042 (8)	0.0815 (6)	0.016*	0.153 (3)
C9B	0.932 (2)	1.059 (2)	0.2133 (12)	0.016*	0.153 (3)
H9B	0.9092	1.1547	0.2278	0.019*	0.153 (3)
C10B	1.0539 (16)	1.0528 (13)	0.1633 (8)	0.016*	0.153 (3)
H10B	1.1190	1.1446	0.1479	0.019*	0.153 (3)
C11B	1.0830 (16)	0.9109 (13)	0.1347 (8)	0.016*	0.153 (3)
C12B	0.982 (5)	0.779 (3)	0.161 (3)	0.016*	0.153 (3)
H12B	1.0049	0.6830	0.1481	0.019*	0.153 (3)
C13B	0.851 (4)	0.788 (4)	0.204 (2)	0.016*	0.153 (3)
H13B	0.7665	0.6950	0.2076	0.019*	0.153 (3)
C16B	1.2738 (12)	1.0334 (9)	0.0393 (6)	0.0224 (18)*	0.153 (3)
H16C	1.1818	1.0880	0.0200	0.027*	0.153 (3)
H16D	1.2972	0.9918	−0.0192	0.027*	0.153 (3)
C17B	1.4473 (13)	1.1485 (11)	0.1044 (7)	0.028 (2)*	0.153 (3)
H37D	1.4800	1.2380	0.0745	0.042*	0.153 (3)
H37E	1.5451	1.1002	0.1156	0.042*	0.153 (3)
H37F	1.4297	1.1822	0.1656	0.042*	0.153 (3)
C18B	1.246 (2)	0.7623 (14)	0.0573 (9)	0.024 (3)*	0.153 (3)
H18C	1.2383	0.7014	0.1084	0.029*	0.153 (3)
H18D	1.3733	0.7919	0.0565	0.029*	0.153 (3)
C19B	1.129 (4)	0.662 (3)	−0.0358 (16)	0.068 (9)*	0.153 (3)
H39D	1.1699	0.5718	−0.0466	0.102*	0.153 (3)
H39E	1.1363	0.7206	−0.0873	0.102*	0.153 (3)
H39F	1.0023	0.6287	−0.0351	0.102*	0.153 (3)

### Atomic displacement parameters (Å^2^)

**Table:**

	*U*^11^	*U*^22^	*U*^33^	*U*^12^	*U*^13^	*U*^23^
I1	0.02157 (4)	0.01510 (3)	0.03178 (5)	0.00740 (3)	0.01155 (3)	0.00642 (3)
O1W	0.0277 (5)	0.0287 (5)	0.0332 (6)	0.0139 (4)	0.0125 (5)	0.0033 (4)
N1	0.0139 (4)	0.0150 (4)	0.0190 (4)	0.0045 (3)	0.0065 (4)	0.0034 (3)
C1	0.0151 (5)	0.0207 (5)	0.0214 (5)	0.0051 (4)	0.0082 (4)	0.0039 (4)
C2	0.0163 (5)	0.0230 (5)	0.0211 (5)	0.0078 (4)	0.0072 (4)	0.0016 (4)
C3	0.0195 (5)	0.0194 (5)	0.0196 (5)	0.0093 (4)	0.0055 (4)	0.0019 (4)
C4	0.0189 (5)	0.0148 (4)	0.0200 (5)	0.0061 (4)	0.0070 (4)	0.0030 (4)
C5	0.0144 (5)	0.0140 (4)	0.0170 (5)	0.0039 (3)	0.0056 (4)	0.0028 (3)
C6	0.0165 (5)	0.0146 (4)	0.0193 (5)	0.0050 (4)	0.0084 (4)	0.0035 (4)
C7	0.0161 (5)	0.0146 (4)	0.0179 (5)	0.0049 (4)	0.0069 (4)	0.0035 (4)
C8	0.0165 (5)	0.0141 (4)	0.0168 (5)	0.0044 (4)	0.0070 (4)	0.0036 (4)
C14	0.0192 (5)	0.0148 (4)	0.0275 (6)	0.0064 (4)	0.0114 (5)	0.0077 (4)
C15	0.0231 (6)	0.0165 (5)	0.0362 (7)	0.0032 (4)	0.0100 (6)	0.0000 (5)
N2A	0.0189 (6)	0.0213 (5)	0.0225 (7)	0.0066 (5)	0.0114 (6)	0.0030 (5)
C9A	0.0178 (10)	0.0138 (5)	0.0158 (10)	0.0042 (7)	0.0062 (8)	0.0022 (8)
C10A	0.0172 (8)	0.0160 (5)	0.0169 (8)	0.0030 (5)	0.0071 (6)	0.0025 (6)
C11A	0.0161 (8)	0.0184 (5)	0.0150 (7)	0.0057 (6)	0.0061 (6)	0.0026 (6)
C12A	0.0204 (17)	0.0155 (5)	0.021 (2)	0.0068 (7)	0.0088 (17)	0.0031 (7)
C13A	0.0179 (13)	0.0137 (5)	0.0203 (17)	0.0051 (7)	0.0076 (13)	0.0045 (8)
C16A	0.0166 (7)	0.0262 (7)	0.0224 (7)	0.0040 (5)	0.0082 (5)	0.0035 (5)
C17A	0.0342 (9)	0.0323 (8)	0.0247 (8)	0.0097 (7)	0.0151 (7)	0.0087 (6)
C18A	0.0256 (10)	0.0258 (8)	0.0205 (10)	0.0122 (7)	0.0120 (9)	0.0036 (7)
C19A	0.0454 (14)	0.0327 (10)	0.0234 (9)	0.0128 (10)	0.0124 (9)	0.0023 (8)

### Geometric parameters (Å, º)

**Table:**

O1W—H1W	0.84 (2)	C12A—H12A	0.9500
O1W—H2W	0.86 (3)	C13A—H13A	0.9500
N1—C1	1.3611 (15)	C16A—C17A	1.520 (3)
N1—C5	1.3672 (15)	C16A—H16A	0.9900
N1—C14	1.4886 (15)	C16A—H16B	0.9900
C1—C2	1.3695 (17)	C17A—H17A	0.9800
C1—H1A	0.9500	C17A—H17B	0.9800
C2—C3	1.3953 (18)	C17A—H17C	0.9800
C2—H2A	0.9500	C18A—C19A	1.532 (4)
C3—C4	1.3796 (17)	C18A—H18A	0.9900
C3—H3A	0.9500	C18A—H18B	0.9900
C4—C5	1.4066 (16)	C19A—H19A	0.9800
C4—H4A	0.9500	C19A—H19B	0.9800
C5—C6	1.4529 (16)	C19A—H19C	0.9800
C6—C7	1.3548 (16)	N2B—C11B	1.369 (11)
C6—H6A	0.9500	N2B—C16B	1.462 (10)
C7—C8	1.4465 (16)	N2B—C18B	1.477 (12)
C7—H7A	0.9500	C9B—C10B	1.379 (15)
C8—C13B	1.39 (3)	C9B—H9B	0.9500
C8—C9A	1.405 (3)	C10B—C11B	1.415 (12)
C8—C13A	1.414 (5)	C10B—H10B	0.9500
C8—C9B	1.42 (2)	C11B—C12B	1.419 (15)
C14—C15	1.5193 (19)	C12B—C13B	1.383 (17)
C14—H14A	0.9900	C12B—H12B	0.9500
C14—H14B	0.9900	C13B—H13B	0.9500
C15—H15A	0.9800	C16B—C17B	1.506 (11)
C15—H15B	0.9800	C16B—H16C	0.9900
C15—H15C	0.9800	C16B—H16D	0.9900
N2A—C11A	1.372 (2)	C17B—H37D	0.9800
N2A—C18A	1.457 (2)	C17B—H37E	0.9800
N2A—C16A	1.462 (2)	C17B—H37F	0.9800
C9A—C10A	1.381 (3)	C18B—C19B	1.484 (16)
C9A—H9A	0.9500	C18B—H18C	0.9900
C10A—C11A	1.416 (2)	C18B—H18D	0.9900
C10A—H10A	0.9500	C19B—H39D	0.9800
C11A—C12A	1.416 (3)	C19B—H39E	0.9800
C12A—C13A	1.382 (3)	C19B—H39F	0.9800
			
H1W—O1W—H2W	105 (2)	C11A—C12A—H12A	119.3
C1—N1—C5	121.81 (10)	C12A—C13A—C8	121.4 (4)
C1—N1—C14	116.17 (10)	C12A—C13A—H13A	119.3
C5—N1—C14	122.02 (10)	C8—C13A—H13A	119.3
N1—C1—C2	121.79 (12)	N2A—C16A—C17A	114.86 (15)
N1—C1—H1A	119.1	N2A—C16A—H16A	108.6
C2—C1—H1A	119.1	C17A—C16A—H16A	108.6
C1—C2—C3	118.30 (11)	N2A—C16A—H16B	108.6
C1—C2—H2A	120.9	C17A—C16A—H16B	108.6
C3—C2—H2A	120.9	H16A—C16A—H16B	107.5
C4—C3—C2	119.53 (11)	N2A—C18A—C19A	114.4 (2)
C4—C3—H3A	120.2	N2A—C18A—H18A	108.7
C2—C3—H3A	120.2	C19A—C18A—H18A	108.7
C3—C4—C5	121.55 (11)	N2A—C18A—H18B	108.7
C3—C4—H4A	119.2	C19A—C18A—H18B	108.7
C5—C4—H4A	119.2	H18A—C18A—H18B	107.6
N1—C5—C4	116.96 (10)	C11B—N2B—C16B	122.6 (7)
N1—C5—C6	119.97 (10)	C11B—N2B—C18B	122.3 (9)
C4—C5—C6	123.06 (11)	C16B—N2B—C18B	115.0 (8)
C7—C6—C5	122.42 (10)	C10B—C9B—C8	122.4 (15)
C7—C6—H6A	118.8	C10B—C9B—H9B	118.8
C5—C6—H6A	118.8	C8—C9B—H9B	118.8
C6—C7—C8	127.43 (10)	C9B—C10B—C11B	120.6 (13)
C6—C7—H7A	116.3	C9B—C10B—H10B	119.7
C8—C7—H7A	116.3	C11B—C10B—H10B	119.7
C13B—C8—C9A	117.3 (10)	N2B—C11B—C10B	120.3 (10)
C9A—C8—C13A	116.9 (2)	N2B—C11B—C12B	123.1 (14)
C13B—C8—C9B	115.7 (12)	C10B—C11B—C12B	116.6 (14)
C13A—C8—C9B	116.6 (7)	C13B—C12B—C11B	121 (2)
C13B—C8—C7	124.1 (10)	C13B—C12B—H12B	119.4
C9A—C8—C7	118.43 (13)	C11B—C12B—H12B	119.4
C13A—C8—C7	124.64 (19)	C12B—C13B—C8	122 (2)
C9B—C8—C7	118.3 (7)	C12B—C13B—H13B	119.2
N1—C14—C15	111.56 (11)	C8—C13B—H13B	119.2
N1—C14—H14A	109.3	N2B—C16B—C17B	114.5 (8)
C15—C14—H14A	109.3	N2B—C16B—H16C	108.6
N1—C14—H14B	109.3	C17B—C16B—H16C	108.6
C15—C14—H14B	109.3	N2B—C16B—H16D	108.6
H14A—C14—H14B	108.0	C17B—C16B—H16D	108.6
C14—C15—H15A	109.5	H16C—C16B—H16D	107.6
C14—C15—H15B	109.5	C16B—C17B—H37D	109.5
H15A—C15—H15B	109.5	C16B—C17B—H37E	109.5
C14—C15—H15C	109.5	H37D—C17B—H37E	109.5
H15A—C15—H15C	109.5	C16B—C17B—H37F	109.5
H15B—C15—H15C	109.5	H37D—C17B—H37F	109.5
C11A—N2A—C18A	121.83 (15)	H37E—C17B—H37F	109.5
C11A—N2A—C16A	120.56 (13)	N2B—C18B—C19B	114.7 (14)
C18A—N2A—C16A	117.61 (13)	N2B—C18B—H18C	108.6
C10A—C9A—C8	122.1 (2)	C19B—C18B—H18C	108.6
C10A—C9A—H9A	118.9	N2B—C18B—H18D	108.6
C8—C9A—H9A	118.9	C19B—C18B—H18D	108.6
C9A—C10A—C11A	121.06 (19)	H18C—C18B—H18D	107.6
C9A—C10A—H10A	119.5	C18B—C19B—H39D	109.5
C11A—C10A—H10A	119.5	C18B—C19B—H39E	109.5
N2A—C11A—C12A	121.9 (2)	H39D—C19B—H39E	109.5
N2A—C11A—C10A	121.11 (16)	C18B—C19B—H39F	109.5
C12A—C11A—C10A	116.9 (2)	H39D—C19B—H39F	109.5
C13A—C12A—C11A	121.5 (4)	H39E—C19B—H39F	109.5
C13A—C12A—H12A	119.3		
			
C5—N1—C1—C2	−2.09 (19)	C10A—C11A—C12A—C13A	−3.2 (7)
C14—N1—C1—C2	178.50 (12)	C11A—C12A—C13A—C8	3.3 (8)
N1—C1—C2—C3	−0.24 (19)	C13B—C8—C13A—C12A	93 (10)
C1—C2—C3—C4	1.65 (19)	C9A—C8—C13A—C12A	−2.0 (6)
C2—C3—C4—C5	−0.85 (19)	C9B—C8—C13A—C12A	9.2 (10)
C1—N1—C5—C4	2.81 (17)	C7—C8—C13A—C12A	−179.1 (4)
C14—N1—C5—C4	−177.81 (11)	C11A—N2A—C16A—C17A	−81.12 (19)
C1—N1—C5—C6	−176.43 (11)	C18A—N2A—C16A—C17A	98.4 (2)
C14—N1—C5—C6	2.94 (17)	C11A—N2A—C18A—C19A	81.4 (2)
C3—C4—C5—N1	−1.35 (18)	C16A—N2A—C18A—C19A	−98.1 (2)
C3—C4—C5—C6	177.87 (12)	C13B—C8—C9B—C10B	−12 (2)
N1—C5—C6—C7	−173.61 (12)	C9A—C8—C9B—C10B	89 (5)
C4—C5—C6—C7	7.19 (19)	C13A—C8—C9B—C10B	−4.8 (18)
C5—C6—C7—C8	−176.54 (12)	C7—C8—C9B—C10B	−177.0 (11)
C6—C7—C8—C13B	8.8 (18)	C8—C9B—C10B—C11B	4 (2)
C6—C7—C8—C9A	−176.71 (14)	C16B—N2B—C11B—C10B	−9.6 (15)
C6—C7—C8—C13A	0.4 (3)	C18B—N2B—C11B—C10B	174.4 (10)
C6—C7—C8—C9B	172.0 (8)	C16B—N2B—C11B—C12B	168 (2)
C1—N1—C14—C15	92.46 (13)	C18B—N2B—C11B—C12B	−8 (3)
C5—N1—C14—C15	−86.95 (14)	C9B—C10B—C11B—N2B	177.1 (12)
C13B—C8—C9A—C10A	−7.1 (16)	C9B—C10B—C11B—C12B	0 (3)
C13A—C8—C9A—C10A	0.7 (3)	N2B—C11B—C12B—C13B	−172 (3)
C9B—C8—C9A—C10A	−90 (4)	C10B—C11B—C12B—C13B	5 (5)
C7—C8—C9A—C10A	178.03 (14)	C11B—C12B—C13B—C8	−15 (6)
C8—C9A—C10A—C11A	−0.7 (2)	C9A—C8—C13B—C12B	6 (4)
C18A—N2A—C11A—C12A	6.8 (4)	C13A—C8—C13B—C12B	−82 (10)
C16A—N2A—C11A—C12A	−173.7 (4)	C9B—C8—C13B—C12B	17 (4)
C18A—N2A—C11A—C10A	−175.31 (17)	C7—C8—C13B—C12B	−179 (3)
C16A—N2A—C11A—C10A	4.2 (2)	C11B—N2B—C16B—C17B	87.0 (11)
C9A—C10A—C11A—N2A	−176.13 (15)	C18B—N2B—C16B—C17B	−96.8 (10)
C9A—C10A—C11A—C12A	1.9 (4)	C11B—N2B—C18B—C19B	93.0 (19)
N2A—C11A—C12A—C13A	174.8 (4)	C16B—N2B—C18B—C19B	−83.3 (19)

### Hydrogen-bond geometry (Å, º)

**Table:**

*D*—H···*A*	*D*—H	H···*A*	*D*···*A*	*D*—H···*A*
O1*W*—H1*W*···I1^i^	0.85 (3)	2.71 (2)	3.5498 (12)	176 (2)
O1*W*—H2*W*···I1^ii^	0.86 (3)	2.75 (3)	3.6055 (13)	171 (2)
C3—H3*A*···O1*W*^iii^	0.95	2.35	3.2072 (19)	150

Symmetry codes: (i) −*x*+1, −*y*+1, −*z*+1; (ii) *x*−1, *y*, *z*; (iii) *x*, *y*+1, *z*.
